# Hidden genes in birds

**DOI:** 10.1186/s13059-015-0724-z

**Published:** 2015-08-18

**Authors:** Tomáš Hron, Petr Pajer, Jan Pačes, Petr Bartůněk, Daniel Elleder

**Affiliations:** Institute of Molecular Genetics, Academy of Sciences of the Czech Republic, Vídeňská 1083, 14220 Prague, Czech Republic

## Abstract

We report that a subset of avian genes is characterized by very high GC content and long G/C stretches. These sequence characteristics correlate with the frequent absence of these genes from genomic databases. We provide several examples where genes in this subset are mistakenly reported as missing in birds.

www.dx.doi.org/10.1186/s13059-015-0725-y

**Electronic supplementary material:**

The online version of this article (doi:10.1186/s13059-015-0724-z) contains supplementary material, which is available to authorized users.

## Main text

A recent paper reported 274 genes as missing in birds but present in the genomes of most other vertebrate lineages [1]. Here, we describe several genes from this list that are, in fact, present in the chicken genome. Importantly, we would like to draw attention to a subset of avian genes characterized by high GC content and multiple long GC-rich stretches. We suggest that the characteristics of these sequences are behind the frequent absence of this gene category from genomic assemblies and other sequence databases. However, the fact is that these genes can, in many cases, be reconstructed from large amounts of “raw” next-generation sequence (NGS) data available from the Sequence Read Archive (SRA) of the National Center for Biotechnology Information (NCBI).

Pursuing our long-term interest in chicken hematopoiesis, we noticed that the gene cluster reported in Figure 2 of Lovell *et al.* [1] next to the erythropoietin receptor (*EPOR*) shows the *LPPR2* gene as missing in birds. However, we already knew that the *EPOR* and *LPPR2* genes existed in the chicken. The sequences of both these genes are in line with the GC-rich characteristics mentioned above. Furthermore, we have examined, though not exhaustively, the list of 274 genes reported as missing in birds [1]. Using mammalian and other vertebrate orthologs of these genes, we analyzed NCBI’s SRA datasets from the chicken and other birds. In this way, we were able to reconstruct two other chicken genes, *MMP14* and *MRPL52*. The sequences of the chicken *LPPR2*, *MMP14* and *MRPL2* genes (Additional file 1) were assembled from multiple pooled RNA-seq datasets from the SRA. Several lines of evidence indicate that these genes are, in fact, the orthologs of corresponding genes in non-avian vertebrates. First, their sequences are absent from the current chicken assembly, or are present only as small fragments in unidentified genomic contigs. Second, phylogenetic analysis (Additional file 2) confirms that they are correctly placed with orthologous genes – not with their closest paralogs, *MMP15* and *LPPR5*. Finally, for *LPPR2* there is at least partial information showing correct synteny in birds. We have assembled the Tibetan ground tit (*Pseudopodoces humilis*) *LPPR2*, which lies on the same 46-kb genomic scaffold [GenBank: NW_005087926] in *P. humilis* as *EPOR* and *SWSAP1*. This is in keeping with gene arrangement in mammals.

**Fig. 1:**
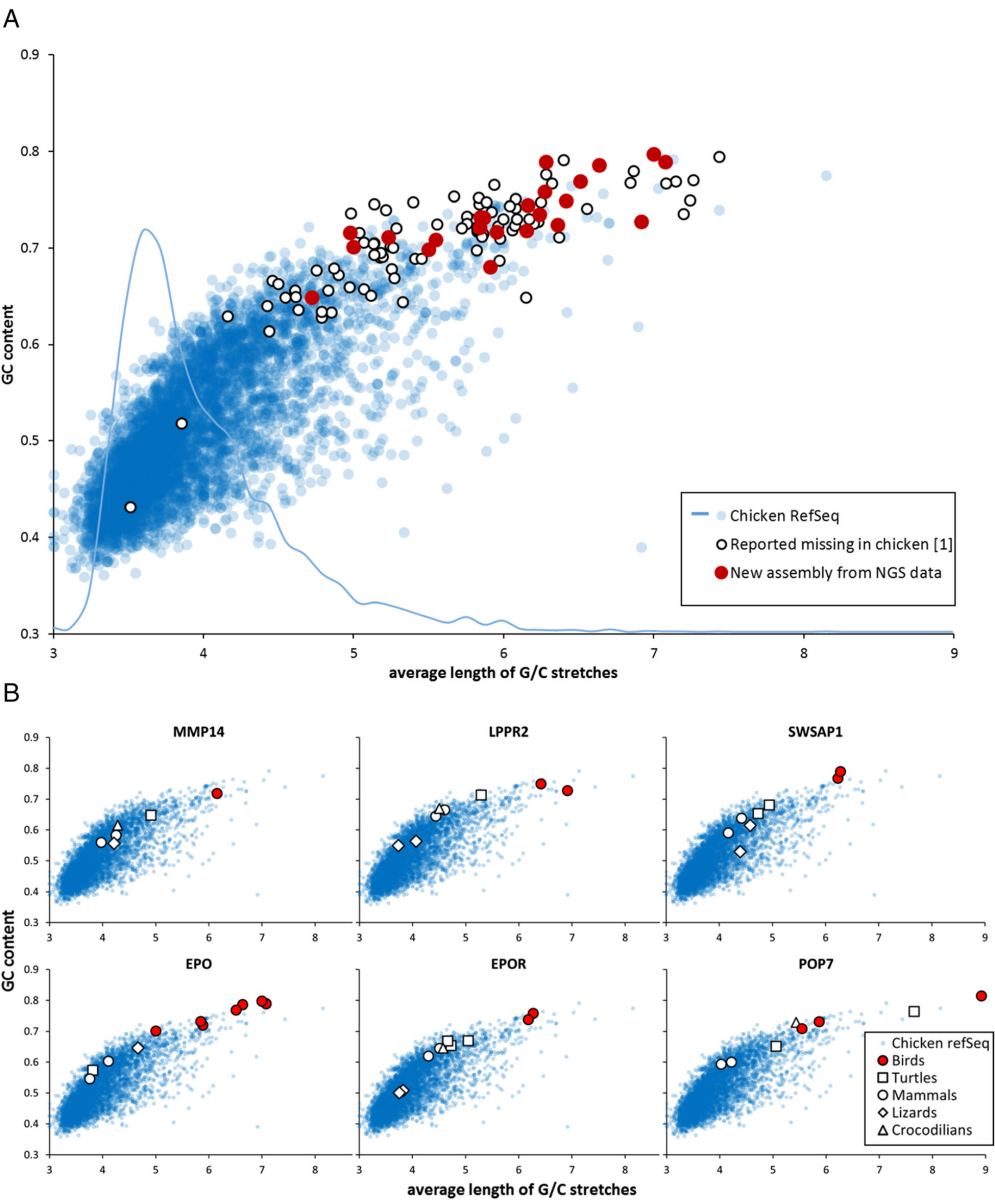
Patterns of GC content and G/C stretches in avian and other vertebrate genes. **a** Dot plot of avian genes, displaying the GC-content and average length of stretches containing G or C nucleotides. G/C-stretch was defined as an undisrupted sequence of at least three consecutive G or C nucleotides. The complete set of approximately six thousand chicken RefSeq genes from the UCSC genome browser database [17] is depicted by blue circles. Only coding sequences longer than 299 nucleotides were analyzed. The set of 86 avian genes reported to be missing in chicken genome [1] are depicted by open circles, and 23 avian genes newly assembled in this study are shown as red circles. Additionally, a histogram showing the distribution of average G/C-stretch length in the chicken RefSeq gene category is depicted by a blue line. **b** Dot plots of selected avian genes, compared with their vertebrate orthologs. GC-content and average length of G/C stretches in coding sequences of chicken *MMP14* and *LPPR2* (reported as missing in birds [1]), and genes from the *EPO* and *EPOR* loci are shown. If available, orthologous genes from other birds, turtles, mammals, lizards and crocodilians are included in the plots. The blue dots show the distribution of chicken RefSeq genes. Sequences of newly assembled avian genes represented in this figure, and GenBank accession numbers of sequences plotted in panel B are listed in Additional file 1 and Additional file 4, respectively

The newly identified chicken *MMP14* and *MRPL2* genes also showed the GC-rich sequence characteristics. To show that this sequence pattern causes persistent problems for correct gene assembly, we analyzed the 89 genes (Supplemental Table 6A in [1]) reported as missing in chicken but present in some other bird species. Using these bird genes as probes, we were able to use the chicken SRA data to assemble several genes from this list (*ALKBH7*, *BLVRB*, *INO80E*, *NDUFB7*, *OPLAH*, *PCP2*, *PET100*, and *SWSAP1*) (Additional file 1). Indeed, as shown in Fig. 1, most of the 89 genes are clear outliers on account of their GC% and G/C-rich stretches. The majority of the 89 genes are from the *P. humilis* genome [2], whose assembly is, in our view, the most complete in terms of coverage of GC-rich avian genes. The distributions of GC% and G/C-stretches in *P. humilis* genes do not differ from those in the genes of other bird species (Additional file 3). Therefore, there is no systematic bias in the sequence composition of the majority of *P. humilis* genes.

Furthermore, we report here for the first time the sequences of chicken erythropoietin (*EPO*) and *EPOR* genes (Additional file 1), which also share the GC-rich sequence characteristics. These genes were absent from nucleotide databases, and it was assumed that avian hematopoiesis did not require *EPO* signaling since primary chicken erythroid progenitors were not EPO-dependent [3–6]. Therefore, the identification of chicken *EPO* and *EPOR* genes allows us to test whether avian *EPO* retains the biological activity it has in other vertebrates.

All these newly assembled avian genes previously considered missing in all birds or in the chicken share similar GC-rich sequence characteristics. GC-rich genes are extremely hard to amplify by PCR, a key step in NGS library preparation [7, 8]. These technical hurdles are presumably behind the absence of this gene subset from genomic databases. In particular, regions of long and concatenated GC-rich stretches cause an extreme decrease in the coverage by NGS reads. Therefore, the assembly of genes in this subset requires multiple large SRA datasets (examples are provided in Additional file 1). We also note that many of the GC-rich stretches are predicted to form DNA quadruplex structures [9]. We can only speculate about the biological determinants behind the presence of the GC-rich sequence patterns. In the genes we have analyzed here, these sequence patterns appear to be conserved in birds but not in other vertebrates. The best example is *EPO*, where we were able to assemble orthologs in several bird species from a wide variety of avian taxons. All avian *EPO* sequences cluster together, while the mammalian and other non-avian *EPO* orthologs have lower GC content (Fig. 1b and Additional file 4). Therefore, the events leading up to this change in *EPO* sequence composition must have occurred in a common ancestor of birds, or there must have been some driving force maintaining this pattern throughout avian evolution. A similar evolutionary trend can be observed in *POP7* (which lies next to *EPO* in vertebrate genomes), *EPOR*, its genomic neighbor *SWSAP1*, and other GC-rich genes reported here (e.g. *MMP14* and *LPPR2*, as shown in Fig. 1b). For these genes, we had only a very limited amount of sequences from outside their coding regions, so their position on avian chromosomes could not be determined. An intriguing possibility is that at least some of these genes reside on avian microchromosomes. The six smallest chicken microchromosomes (chromosomes 33-38) do not have any sequence representation in the chicken genome assembly [10]. Sequence information for the larger chicken microchromosomes is also fragmentary; they have, however, been reported to have higher GC content than macrochromosomes [11, 12]. In addition, avian microchromosomes contain various types of short microsatellite repeats [13–16]. The extensive presence of these repeats is a typical feature that we observe in introns in the GC-rich gene subset.

## Conclusion

We report the existence of avian genes with strongly biased GC patterns. These genes have been underrepresented in genomic databases, probably due to technical obstacles to genomic library preparation. In addition to identifying chicken *EPO* and *EPOR* loci, we analyzed the gene set reported as missing in birds [1] and found additional examples of such genes. Our examination of the genes listed in Lovell *et al*. [1] was not exhaustive, so among the avian genes absent from current databases several more can be expected to be assembled from SRA data. Nevertheless, the vast majority of the genes reported in Lovell *et al*. [1] are probably really missing in birds, and their article includes a detailed discussion of the evolutionary aspects of this phenomenon. The existence of an underrepresented GC-rich gene subset was originally suggested in the 2004 report on the chicken genome sequence [12]. Here, we present detailed examples of such genes, which present an analytical challenge from both technical and evolutionary perspectives.

## Additional files

Additional file 1:
**Sequences of newly assembled avian genes.** The list includes fourteen chicken genes, three genes from *P. humilis* and six genes from other bird species. For the assembly of the chicken genes, we used mostly the following large datasets from the NCBI SRA: i) ERP003988, SRP026393, SRP033603, and SRP014719, representing approximately 1.1 terabases (Tb) of sequence data from RNA-seq studies, and ii) SRP034930, SRP042641, SRP040477, and SRP040256, representing approximately 1.4 Tb of genomic data. The downloaded sequences were assembled using either CLC genomics workbench 6.5.1 (http://www.clcbio.com) or DNASTAR Lasergene 10.0.0 (http://dnastar.com). When the coding sequence (CDS) of a gene could not be assembled due to low or missing coverage by sequence reads, it was indicated as 5′/3′ truncation or internal gap. The sequence of chicken *EPO* was verified by PCR amplification from chicken cDNA (primers 5′- GCAGCGGCCGCAATGAGAC and 5′- GGGTCACCGCCAGTGCCG) and submitted to Genbank under accession number [GenBank:KR063574]. Additional file 2:
**Phylogenetic analysis of LPPR2, MMP14, and MRPL52.** The three newly assembled genes were analyzed together with orthologous sequences from other vertebrates, and with their closest paralogs, if those were available. The list of GenBank sequences used in the alignment is given bellow. Amino acid sequences were aligned with the MUSCLE algorithm and uninformative regions were removed using trimAl v1.4 software. The maximum likelihood (ML) phylogeny was constructed in MEGA 6.06 software, using the JTT substitution model, Nearest-Neighbor-Interchange ML heuristic method and otherwise default parameters. Support for the ML tree was assessed by 200 nonparametric bootstrap replicates. Bootstrap values higher than 0.95 are shown. Red asterisks mark the newly assembled avian genes. Scale bars show the number of amino acid substitutions per site. Additional file 3:
**Comparison of GC-content and the presence of G/C stretches in genes of three avian species.** The GenBank RefSeq datasets for chicken (*Gallus gallus*), Tibetan ground tit (*P. humilis*), and zebra finch (*Taeniopygia guttata*) were analyzed. Only coding sequences with length greater than 299 nucleotides were included. GC-stretch was defined as in legend to Fig. 1. Both dot plots and histograms of G/C stretch average lengths are shown. The histograms have similar shapes but not heights, because different number of genes is annotated in the three avian species. Additional file 4:
**List of avian genes and their vertebrate orthologs used in Fig.** **1b** **.**

## Abbreviations

NGS: Next-generation sequencing
NCBI: National Center for Biotechnology Information
SRA: Sequence read archive
EPOR: Erythropoietin receptor
EPO: Erythropoietin

## References

[1] Lovell PV, Wirthlin M, Wilhelm L, Minx P, Lazar NH, Carbone L, Warren WC, Mello CV (2014). Conserved syntenic clusters of protein coding genes are missing in birds. Genome Biol.

[2] Cai Q, Qian X, Lang Y, Luo Y, Xu J, Pan S, Hui Y, Gou C, Cai Y, Hao M (2013). Genome sequence of ground tit Pseudopodoces humilis and its adaptation to high altitude. Genome Biol.

[3] Beug H, Steinlein P, Bartunek P, Hayman MJ (1995). Avian hematopoietic cell culture: in vitro model systems to study oncogenic transformation of hematopoietic cells. Methods Enzymol.

[4] Dolznig H, Bartunek P, Nasmyth K, Mullner EW, Beug H (1995). Terminal differentiation of normal chicken erythroid progenitors: shortening of G1 correlates with loss of D-cyclin/cdk4 expression and altered cell size control. Cell Growth Differ.

[5] Hayman MJ, Meyer S, Martin F, Steinlein P, Beug H (1993). Self-renewal and differentiation of normal avian erythroid progenitor cells: regulatory roles of the TGF alpha/c-ErbB and SCF/c-kit receptors. Cell.

[6] Schroeder C, Gibson L, Nordstrom C, Beug H (1993). The estrogen receptor cooperates with the TGF alpha receptor (c-erbB) in regulation of chicken erythroid progenitor self-renewal. EMBO J.

[7] Aird D, Ross MG, Chen WS, Danielsson M, Fennell T, Russ C, Jaffe DB, Nusbaum C, Gnirke A (2011). Analyzing and minimizing PCR amplification bias in Illumina sequencing libraries. Genome Biol.

[8] Ross MG, Russ C, Costello M, Hollinger A, Lennon NJ, Hegarty R, Nusbaum C, Jaffe DB (2013). Characterizing and measuring bias in sequence data. Genome Biol.

[9] Menendez C, Frees S, Bagga PS (2012). QGRS-H Predictor: a web server for predicting homologous quadruplex forming G-rich sequence motifs in nucleotide sequences. Nucleic Acids Res.

[10] Griffin D, Burt DW (2014). All chromosomes great and small: 10 years on. Chromosome Res.

[11] Costantini M, Di Filippo M, Auletta F, Bernardi G (2007). Isochore pattern and gene distribution in the chicken genome. Gene.

[12] International Chicken Genome Sequencing C (2004). Sequence and comparative analysis of the chicken genome provide unique perspectives on vertebrate evolution. Nature.

[13] Deryusheva S, Krasikova A, Kulikova T, Gaginskaya E (2007). Tandem 41-bp repeats in chicken and Japanese quail genomes: FISH mapping and transcription analysis on lampbrush chromosomes. Chromosoma.

[14] Ishishita S, Tsuruta Y, Uno Y, Nakamura A, Nishida C, Griffin DK, Tsudzuki M, Ono T, Matsuda Y (2014). Chromosome size-correlated and chromosome size-uncorrelated homogenization of centromeric repetitive sequences in New World quails. Chromosome Res.

[15] Krasikova A, Fukagawa T, Zlotina A (2012). High-resolution mapping and transcriptional activity analysis of chicken centromere sequences on giant lampbrush chromosomes. Chromosome Res.

[16] Shang WH, Hori T, Toyoda A, Kato J, Popendorf K, Sakakibara Y, Fujiyama A, Fukagawa T (2010). Chickens possess centromeres with both extended tandem repeats and short non-tandem-repetitive sequences. Genome Res.

[17] Rosenbloom KR, Armstrong J, Barber GP, Casper J, Clawson H, Diekhans M, Dreszer TR, Fujita PA, Guruvadoo L, Haeussler M (2015). The UCSC Genome Browser database: 2015 update. Nucleic Acids Res.

