# Supplementary material for: Hidden genes in birds
Source: Genome Biol. 2015 Aug 18;16(1):164. doi: 10.1186/s13059-015-0724-z (PMC4539667; doi:10.1186/s13059-015-0724-z)
Supplement: Additional file 1: — Sequences of newly assembled avian genes. The list includes fourteen chicken genes, three genes from P. humilis and six genes from other bird species. For the assembly of the chicken genes, we used mostly the following large datasets from the NCBI SRA: i) ERP003988, SRP026393, SRP033603, and SRP014719, representing approximately 1.1 terabases (Tb) of sequence data from RNA-seq studies, and ii) SRP034930, SRP042641, SRP040477, and SRP040256, representing approximately 1.4 Tb of genomic data. The downloaded sequences were assembled using either CLC genomics workbench 6.5.1 (http://www.clcbio.com) or DNASTAR Lasergene 10.0.0 (http://dnastar.com). When the coding sequence (CDS) of a gene could not be assembled due to low or missing coverage by sequence reads, it was indicated as 5′/3′ truncation or internal gap. The sequence of chicken EPO was verified by PCR amplification from chicken cDNA (primers 5′- GCAGCGGCCGCAATGAGAC and 5′- GGGTCACCGCCAGTGCCG) and submitted to Genbank under accession number [GenBank:KR063574]. [file 13059_2015_724_MOESM1_ESM.docx]

>Gallus gallus - lipid phosphate phosphatase-related protein type 2 (LPPR2), CDS - 3' truncated

ATGGCCGCCCCCCGGCGGGAGGTGAAGAGGAGCAGTGGGATCGTGCCGTGCTTCCTCTGCGTGGAGCTGGGTATTTTGGGGGGCACGGCGGCCCTCGCCTACCAGCTGGAGTTCACCGACGCCTTCCCTGTGCACGAGGGGGGGTTCTTCTGCCGGGACCCCCAATATGGGCGCCCCTATCCCGGCCCCCCCGCCAACAGCCGCGCCCCCCCCGCCCTCGTTTACTCTCTGGTCACCGCCGTGCCCACCCTGACCATCGTGGTGGGGGAGCTGGCGGGGCGGCTGGGGGGGGCGCGGGGGGGCCGTGACCCCACAATTCTGTGGGGGGAGTGCTGCTATTTGGGGGCCCCACTGCGGCGGCTGCTGCGCTTCCTGGGCGTCTTCTCCTTCGGCCTATTGGCCACGGCCATCTTCGCCAACGCGGGGCAGGTGGTGACGGGGACCCCCGCCCCCCATTTCTTGGCCGTGTGCCGCCCCAACTACACGGCGTTGGGCTGCGCCCCCCCCCGCCCGGCCGCCCCCCCCCACTTCGTCACAGCCGGGGGGGCCGCGTGCGCCGGTGACCCCCCCTTGGTGGCCGCTGCCAGGAGGGCTTTCCCGTGTAAAGAGGCGGCGTTGGGGGCCTATGGGGGGGCGTACGCTGCGCTGTACGTGACGTTGGCGTGGCGGGGGGGGGGCTCCCGGCTGGCCAAACCCGCGGCCGTGTTGGCGTTGTGCGCCCCCCCCTTTTTGGTGGGGGCCGTCCGGGTGGCGGAGCACCGCAACCATTGGGCCGATGTGTTGGCCGGCTTCGTCACCGGCGGAGCCATCGCTGCCTTCCTGGTGAGCTGCGTGGTGGGCAACTTCCAGTGCAAGGGGGGGCCGCTGGGGGGGGGCTCAGGGGGGGGGGCGCCCCMCRGAGCCCCCCCAGAAGTGCCCCGACCTCAACCCCCCCTGGAGGAGCTGAGCGTCACCCAGACGCGCCGTGCTGAGTTCCCGGCCGTCACC

>Pseudopodoces humilis - lipid phosphate phosphatase-related protein type 2 (LPPR2), CDS - 3' truncated

ATGGCCCCCCCCCGGCAGGACAAGCGCGGCTCCATCGTCCCCTGCGTGCTCCTGGTGGAGGTGGGGATTTTGGGGGGCACGGCAGCCCTCGCCTACCAGCTGGAGTTCACCGACGCCTTCCCGGTGCACGTGGGGGGTTTCTTCTGCCGGGACCCCGATTTTGGGCGCCCCTACCCGGGACCCCCCGGGCTGAGCCGGGCCCCCCCCGCCCTGGTCTATGCCCTGGTCACCGCCGTGCCCGCCCTCACCATGGCGGTGGGGGAGCTCCTGGGCCGTTTGGGGGGGTCCCGGGGGGGTCGCGCCGCCCCCCCGCGCTGGGCCCGGAGCTGCGAGCGGGGGGCGCCGCTGCGGCGCCTGCTGCGATTCCTCGGTGTGTTCTCCTTCGGCCTCCTCGCCACCGCCATCTTCTCCAACGCCTTCCAGGTGGTCCTGGGGACCCCCGCCCCCCATTTCCTGGCCGTCTGCCGCCCCAACTACAGCGCCCTGGGCTGCGCGCAGCCCCCCGGGACCCCCGCGACCCCTCCCCAATTCGTGCCCCCGGGGGGCTCCCCCTGCTCCGGGGACCCCCCGGCMGTGGCCGCGGCCCGGAGGGATTTCCCCTGCAAGGAGGCGGCGCTGGGAGCCTACGCGGGGGCGTTCGCCGGGCTGTACGTGACCCTGGCCTGGCGGGGGGGGGGGTCTCGCCTGGCCAAGCCCGCGGCGGTTTTGGGGTTCGCGGCCCCCCCGTTCCTGCTGGGGGCTCTGCGCGTGGCCGAGCACCGCAACAGCTGGGGAGGGGTCCTGGGGGGCTTCCTGTGCGGCACCGCCATCGCCGCCTTCCTGGTCACGTGCGTGGTCGGAAACTTCCAAATCCCCCC

>Gallus gallus - matrix metallopeptidase 14 (membrane-inserted) (MMP14), CDS

ATGGCGCCCGCTCTGCTGCTGCTGCTCCTCTGCTGCGCCGCCCCCCCCCCCGCCGCCGCCTTCCGGCCCGAGGCGTGGCTGCAGCAGTACGGCTATCTGCCCCCCGGCGACCTCCGCGCCCACCCCCCCACTTCGGCCCATTCGGTATCGGCCGCGTTGGCCGCCATGCAGCGCTTCTACGGGCTGCGCGTCACCGGAAGTGTCGACCCGGAAACGCTGCGGGCCATGAAGCGCCCCCGCTGTGGGGTCCCGGACCGCTTTGGGGCGGAGGTGAAGGCCAACGTGAGGCGCCGCCGTTACGCCATCCAGGGCTCCAAGTGGGAGCAGCGCGACATCACCTTCTGCCTTCAGAACCACACCCCGAAGGTGGGGGAGGCGGCCACCCGCGCTGCCATCCTCCGCGCCTTCGGGGTGTGGGCGTCCGTCACCCCACTGCGCTTCCGGGAAGTGCCCCCCGGCGCCGCCCCCCCCGCCGACATCGTCCTCTTCTTCGCCGAGGGCTTCCACGGCGACAGCTCCCCCTTCGACGGCGAGGGGGGGTTCCTGGCCCACGCCTACTTCCCCGGGCCCCACATCGGGGGGGACACGCACTTCGACGGCGCCGAGCCCTGGACCACGCGCAACGACGACCTCAGCGGTCACGACGTGTTCCTGGTGGCGCTGCACGAGCTGGGCCACGCGCTGGGCCTGGAGCACTCCAGCGACCCCTCGGCCGTCATGGCGCCCTTCTACCAATGGATGGACACCGCCCCCTTCGTGCTGCCCGACGACGACCGCCGCGGCATCCAGCAGCTCTACGGGCCGGGTCCCAACATGCCCCCCCCGGACCCCCGCGGCACAGCGCTGCCCCACGACCCCGACCGGCCGCCCCACGGCCCCCCCTATGGGCCCCGCATCTGCGACGGCGGCTTCGATACCATCGCGGTGCTCAGGGGGGAGATGTTCGTGTTCAAGGAGCGGTGGCTGTGGCGGCTGCGGGAGCGCCGGGTGCTGCCCGGTTACCCCCTCCCTATGGGGCAGCTGTGGCCCGGACTGCCCCACAGCATCGACGCCGCCTATGAGAGGAAGGACGGCAAGTTCGTCTTCTTCAAAGGCGGGCGGCAGTGGGTGTTCTCGGAGGCGGCGCTGCAGCCGGGCTTCCCGCGCGCTCTGCCGGACGTGGGCCGGGGGCTGCCGGAGCGCATCGACGCCGCGCTGCTGTGGCTGCCCAGCGGGGCCACGTACCTCTTCCGGGGCGACAAGTACTACCGGTTCAATGAGGAGACGGAGTCGGTGGACCCCGATTACCCCAAAAGCATTTCCGTGTGGGGCGGCGTCCCCGAATCACCCCAAGGAGCATTTATGGGGTCGGATGACGCCTACACGTACTTCGTGAAGGGCTCCCGCTATTGGCAGTTCGACAACCGCCAGCTGCGCGTCACCCCGGGTTACCCCAAATCCCTGCTCCGCGATTGGCTGGGCTGCCCGGAGCCCCGCCCACCACCCCGCCCTGGCCCCGCCCCTTCCTCTTCCCCGCCGGAAACGGGCGCCGGGGGGGGGGGAGGGGAAACGGAAGTCATCGTCATCGAAGTGGGCGGGGAAGGGGCGGGGCCCGGAGCGGTGGCCACGCCCCTGGCGCTGCTGGGGGGGGCCGGGGGGCTGCTGGCGGCCGTGCTGTGGTTCCGCCGCCGGGGGGCGCCCAAGAAACTGCTGCGCTGTCAGCGCTCCCTCCTGCCCCGCGTTTAG

>Gallus gallus - mitochondrial ribosomal protein L52 (MRPL52), CDS

ATGGCGGCGCGCAAAGCGCTGCGGATCGCGGAGCTCCGCTCCCTCTCTGCCCGCCCCATTCCTGCGGCCCCACAGCGCATCGGCCAATGGCGCGTCAGCAAAGGCTTGGCCCCCGGCAGTTCGGGCTATGGGCCCCTCCGTGACCTCCCTGATTGGTCCTTTGTGGATGGCCGCCCAGCTCCCCTGTGGGCGGGGCAGCTGCGCCGTCGCCATGACAACGAGGAAGTTGCCCGCCGCGCCGTCGCTCTCATCCAATCAATGGACGCCGCTCGGGAGAGGGGGCGGGGCTTATCCCTCAAGCCCCGCCCCTCGCTGCGCCCCAAAGGCTCCGCCCCCAAATCAATAAAAGACGAATGA

>Gallus gallus - erythropoietin receptor (EPOR), CDS

ATGGCGGCTCCGGGGGTGCTGCTGGCGCTGGGGGGGGTCCTGGCGGCCGCGGGGGGGGGCGCTGAGACCCCCATGGACTTCGAGGTGGAAGCGGCGGTTCTGCAGGCGGAGGAGGCGGCGGACCCGAAGTGCTTCTCGCGGCGGCTGCACGACCTGCTGTGCTTCTGGGACAGCGACGGCCCCCCCGACCCGCAGCTCTTCCAGATGCACTTCCGCCTCGATTCGGATCCGTGGCAGCGCTGCCCGCTGAGCGCGGCGCGGCGGTCGGCGCTCCGTTCGCGCTTTTGGTGCTCCGTCCCTCCGAGCGCCGCCGTCGCCTTCGTGCCGTTGGAGCTGCGCGTTGTGCGCGCGCACAGCGGGGCCGCCGTGCACCGCCGGACGGTGTTCGTGGAGCGCGTGGTGCTGCTGGCCGCCCCGCACAACGTATCGGCGCACGCGGGCGGCGCTCCGGGCGCGCTGTGCGTTCGTTGGCAGCCGCCGCCCAACCCCTACTTGGAGTCGAGCCTCACCTACGAGCTGCTGCTGCGCGCCCCCGGGACGGCGCCGCGCACGGTGGGGGTCCCGGTGGGCCGCCTGGAGCAGCGGGTGGGGGCTCTGAGGGGTCGCACCCCATACACCGTCCGGGTCCGCGTCCGCCCCGACGGGCTGAGCTACGGCGGCTATTGGAGCCCGTGGTCCGAACCCATCACTGCCGTCACCGCCCCCGATGTGGACCCGGTGACGGTGGGGCTGTCCTCTCTGCTGGCGCTGCTGCTGCTGGGGCTGGCAATGCTCGCGCTGCTCGGACAGCGGCGGAAGCTGCAGGAGAAGCTGTGGCCGCCCGTGCCCGGCCCTGAGAGGGAATTCGAGGGGCTCTTCAGCGCCTATGGGGGCAATTTTCAGCTCTGGCTGTACCAAGGGGTGGTGGAGCCCTGGAGCCCCCCCGGAGGCACCCCGGAAGCCGAGGAACAGCCCAGTGCCGTGGAAGAGGTGGGGCCCCCCCCGGGCAAAGAGCCCCCCCCGGGGACCCCCCCGTCTGCCCCCCCTTCGGCCCCCCCCAGCGGCCCCTCGCCCGCCTCCAGCTTTGAGTACACGCTGTTTGACCCCGGCTCGGCCCTGCTCTGCCCCAGGGGGCACCCCCAAATCGCCCCCCCCCACGATCCCCCCGGCGGCCCCTACGCCAACCTGGCCCCCCCCCACAAAGGGCCCCCCCCGCCCGAGGAGGGGACCCCCAAAGAAACCCCCCACGACAGGAGACCCCCACGGGAGCTTCCGTGCAATGGGAACCCCCCCGGGACCCTCCTGGCGTTGGGGCCCCCCCCAATGCCCCCCCCCTACGTGCTGTGCTCTTAA

>Pseudopodoces humilis - erythropoietin receptor (EPOR), CDS

ATGGCGGGGCTGCTGCTGCTGGGGGCGCTCCTGGGGGGGCTCCTGGGGGCGCCCCCTCGGATTTGGGGGGGCCCGGGGGGGGTCCTGGGGCTGCCCGGGGGCTCCGAGACCCCCCCGGAGCTGGAGGAGGAAGTGGCCATTGTCCAGCGGCAGGACCCCGAGCCCCCGCACTGCTTCTCGCGGACCCTGCACGACCTGAGCTGCTTCTGGGACAGCCCCGGGCCCGCCGAGCCGAGCCGCTTCCGCTTCCAGTTCCGCCTCGAGCAGGACCCGTGGCAGGAGTGTCCCCTCAGTGTCACCGCGCTGCCCCGGGGGTCTCGTTTCTGGTGCTCGCTGCCCCCGGCCGCCACCGTCACCTTCGTGCCCCTCGAGCTGCGCGTGCTGCCCGCGACCCCCGGGACCCTCCCCGAGACCACCGGGACCACCGAGACCCCCCCCGGGACCCCCACCGGGACCCCCGCGACCACCGGGACTCCCGGGACCCCCACCGGGACCACCGAGACCCCCCCTGGGACCACCGAGACCCCCCCTGGGACCACCGGGACCCCCGCGACCCCCGGGACCCCCATCGGGACCACCGGAATTCCTCCCGCGACCACCGGGACCACCCCCAAAACCTCTGAGACCCCCGGAACACCCCCCGGGACCACCGGAACTCCTCCCGCGACCAACGAGACCACCGGGACCCCCCCCGGGACCACCGGGACCCCCTCCAACACCTCTGGGACCCCCGGGAGCCCCCCCCAAAGGACGGAGCCCCCTCCCCCCCTGTTCCAGCGGATCCTTTTCATCGACCAAGTCGTGCTGCCGGGACCCCCCCAGAACGTGTCGGTGTCGGCGGGGGGGTCGCGGGGGGAGCTGTGCGTGCGCTGGGCCCCTCCCCCCGGGCCGTACCTGCACTCCAGCCTCATCTTCCAGCTGGCCCTGAGCCCCCCCGAGGGACCCCCAAAAACGGGTGGGGGTCCCGGCGGGCCGGCGCGAGCAGGGGGTCGGGGGTCTCCGGGCCAGCACCGAGTACTCGGTGCGAGCCCGGGCCCGGCCCGACGGGATCAGCTACAGCGGCTTCTGGAGCCCCTGGTCCCCTCCCCGCAGTGCCACCACCCCCCCCGTGCTGGACGCGGTGACGCTGGGACTGGCGAGCCTGCTGGTGCTGCTGCTGCTGGCACTGGGAGCACTGGGACTGCTGGGACACCGCCGGACCCTGCGGGCCAAGCTGTGGCCGCCGGTGCCGGGCCCGGAGCGAGAGTTCGAGGGGCTCTTCAGCGCCTACGGCGGCAACTTCCAGCTGTGGCTGTGCCAGGGCCCGGGCTCTCCCTGGGCGCCCCCCGCGCCCCCCCCCTTGGAGGCCGAGGACGCCGTGGAGGAGGTGGGGGGGGGTCCGGGGGTTCCCCCCTCGCCGGAGCCGCCCCTCCCCCCCGGGGCTGAGACCCCCCCCCAGCCCCGCGACCCCCCCGGGGACCCCTCCCCCTCCCCCAGCTTTGAGTACACGCTGTTCGAGCCCGGCTCCGCCCTGCTCCGCCCCTCCCCCCGCGGCCCCGCCCCCTACGCCAACCTCGCCCCGCCCCCCAAGGGCGCCGAGCCCGCGGGGGCGGGGCAAGGCCCTCATTACGTCATCTGCTCCTGA

>Gallus gallus - erythropoietin (EPO), CDS

ATGGATGTCAATGGGGCCGGGCTGTGTGCGGTGCTGCTGCTGCTGCTGCTGCTGCGGGGGGGGGGGGGGGGGCGCCCCGACGGCCCCCCCTCACTGTGTGACCCCCGAGTGATGGAGAGGTTCATCCGGGAGGCGCGCGACGCTGAGAGGGGGATGGTCGGCTGTGGGCGGCGCTGTGATCTCCCCGAGGCGGTGGCCGTCCCGGACCCCGGCGTCAGCTTCAGCGAATGGCAGCGCATGGATGTGGGGGCTCGGGTTCGGGCGGTGCTGGGGGGCCACGCGGTGTTGGTGGCTGCGGTGCTGCGGGCGCGGGAGCTGCTGAGCGACCCCCAACTCCGACCCACACTGGATCTGATCTATGGGGCAGCACGGAGTCTGGCACACCTGCTGAGGGGGGTGGTCAGCCCGCCCACCCCCACCCCCACCCGCACCCCCCACTCTCCCACCCCCACCCCCTTTTCTCCCACCCCCTCTTCTCCCACCCCCTTTTCCCCCCCTTCCTCCCCCCACTCCGCCCCCCCCCCGCCCCCTCCCCCCCCCCAGGTGAGGACCCTCAGCCGCCTTTTGGGGGTCCACAGCGCTTTCCTCCGTGGCAAAGTCCGGCTGCTGCTCATTGATGTCTGCACCCCGGTGTCCCCCCCCCGGCACTGGCGGTGA

>Pseudopodoces humilis - erythropoietin (EPO), CDS

ATGGGGGCGGCGGGGCTGTGCGTGCTGCTGCTGCTGCTGTTGGGGGTCCCGGGGCGCCCCCAGCCCCCCCCCGAGGGCCCCCCCTCGCTCTGCGACCCCCGAGTGATCGAACGCTTCATCCTGGAGGCCCGCGACGCCGAGCGGGGGGCGGCCGGGTGTGGCCCCCACTGTGACCTCCCCGAGCCCATCGCTGTCCCCGACCCCGGCGTCAACTTCAACCTGTGGCAGAGCCTGGACGCGGGGTCCCGGGCGCAGGAGGTGGCGGCGGGACAGGCGGCGCTGGCGGCCGCGGTGCTGCGGGCGCGGGAGCTGCTCCGGGACCCCCGAGTGCGCCCCAGCCTGGACCGCGCCTACGGGACCGCGCGGAGCCTGGCGCGGCTGCTGCGGGGGCTGCCCGCGCCGGCTCCGCCCCTGCCCTCGCCCCCTGCCCCGCTGCGGGTCCGGACCCTCCCCCGGCTCCTGGGGGTCCTGAGCCGCTTCCTGCGGGGGAAGGTCCGGCTCTTCCTGGCCGACACCTGCCCGCGGTGA

>Ficedula albicollis - erythropoietin (EPO), CDS - 5' truncated

CCCCCCCCGAGGGCCCCCCCTCGCTCTGCGACCCCCGAGTGATCGAGCGCTTCATCCTGGAGGCGCGCGAGGCCGAGCGGGGGGCGGCCGGATGCGGCCCCCCCTGTGACCTCCCCGAGCCCCTCGCTGTCCCCGACCCCGGCGTCAACTTCAACCTCTGGAGGAGCCTGGACGCCGGGGCCCGCGCCCGGGAGGTGGCCGGTGGCCAGGCGGCGCTGGTGGCCGCGGTGCTGCGGGCGCGGGAGCTGCTGCGGGAGCCGCGGGTCCGGCCCAGCCTGGACCGGGCGTACGGAGCGGCGCGGAGCCTGGCGGGGCTGCTGCGGGGGCTGCCGCGGGCCCCGCCCCCCGCCCCCGTTCGGGTCCGGACCCTCCCGCGGCTCCTGGGGGTCCTGAGCCGCTTCCTGCGCGGGAAGGTGCGGCTCTACCTGGCGGACACCTGCCCGCGGTGA

>Haemorhous mexicanus - erythropoietin (EPO), CDS - internal gap marked by N's

ATGGGGGCGGCGGGGCTGTGCGCGCTGCTGCTGCTGCTGTTGGGGGTCCCGGGGCGCCCCCAGCCCCCCCCCGAGGGCCCCCCCTCGCTCTGCGACCCCCGAGTGATGGAGCGCTTCATCCTGGAGGCGCGCGACGCCGAGCGGGGCACGGCCGGGTGTGGCCCCCACTGTGACCTCCCCGAGGCCGTCGCTGTCCCCGACCCCGGCGTCAACTTCAACCTGTGGCGGAGCCTGGACGCGGGGTCCCGCGCGCAGGAGGTGGCCGGGGGCCAGGCGGCGCTGGCGGCCGCGGTGCTGCGGGCGCGGGNNNNNNNNNNNNNNNNNNNNNNNNNNNNNNNNNNNNNNNNNNNNNNNNNNNNNNNNNNNNNNNNNNNNNNNNNNNNNNNNNNNNNNNNNNNNNNNNNNNNNNNNNNGCCCTCGCCCCCCGCCCCCGTGCGGGTCCGGACCCTCCCCCGGCTCCTGGGGGTCCTGAGCCGCTTCCTGCGGGGCAAGGTGCGGCTCTACCTGGCCGACACCTGCCCGCGCTGA

>Coturnix japonica - erythropoietin (EPO), CDS - 3' truncated

ATGGGCGGCGATGGGACCGGGCTGTGTGCGGTGCTGCTGCTGTTGCTGCGGGGGGGGGGCGGGGGGCCCATGGCCCCCCCCCCCCCCTCATTGTGCGACCCACGAGTGATGGAGAGGTTCATCAGAGAGGCCAGGGACGCTGAGAGGGGGATGGCCGGCTGTGGGTCGCGCTGTGATCTCCCCGAAGCTGTGGCCGTCCCAGACCCCGGCGTCAGCTTCAGTGAATGGCAGAGCATGGATGTGGGGGCTCGGGCTCGGGTGGTGCTGGGGGGCCACGCGGTGTTGGTGGCTGCGGTGCTGCGAGCGAGGGAGCTGCTGAGCGACCCCCACCTCCAACCCACGCTTGATCGGATCTATGGGGCAGCACGGAGCCTGGCACACCTGCTGAGGGGGGTGGTCAGCCCG

>Anas platyrhynchos - erythropoietin (EPO), CDS - internal fragment

CCGCGTGCTGGAGAGATTCATCCTGGAGGCGCGCGACGCGGAGAGGGGGCTGGCCAGCTGCGGCCCCCCCTGCGACCTGCCCGAGGCCGTGACCGTCCCCGACCCCGGCGTCAACTTCAACGACTGGCGGCGGATGGACGTG

>Aquila chrysaetos canadensis - erythropoietin (EPO), CDS

ATGGGGGCGGTGGGGCTGTGTGCGCTGGTGCTGATGCTCTTGGGGGTCCCGGGGCGCCCCGACGGCCCCCCCTCGCTCTGTGACCCCCGAGTGATGGAGAGGTTCATCCTGGAGGCCCGCGACGCTGAGAGGGGGCTGGCCGGCTGCGGCCCCCCCTGTGACCTGCCCGAGGCGGTGGCCGTCCCCGACCCTGGCGTCAACTTCAACGACTGGCAGCGGATGGACGCGGGGGCGCGGGCGCGCGAGGTGGGGGGGGGGCAGGCGGTGCTGGTGGCCGCCGTCCTGCGGGCGCGGGAGCTGCTGCCGGACCCCCGCCTGCGCCCCACCCTCGACCGGGCCTACAGCGCGGCCCGCAGCCTGGCCCGCCTGCTGCGGGGGGTCCCCACCCCGGACCCCCCCCGAGATGACCCCCCCCCCAGGTTGCGGGTGCGGACCCTGGCCCGGCTGCTGGGGGTGCACAGCAGCTTCCTCCGCGGGAAGGTCCGGCTCTTCCTCGCCGACGCCTGTCGCCGATGA

>Gallus gallus - SWIM-type zinc finger 7 associated protein 1 (SWSAP1), CDS

ATGGCGGCAGCGCTGGAACGGGCGCTGGGCCCGGCTGGGCCGGAAGCGGACGCGTCCGTGCCGGAGGCGCCGCTGTTGGTGCTCGGCCCGGCGGGCTCGGGCCGCACGGCGCTGCTGCTGCGGGCGGCGTTGGCGGGAGGAGGGGACGGGCCCCGCGCTCTCTTCTTAGCGCCCAGCGCTCCCTCCCGACTCCCAGACGGCGGCGGCGGTGACCCGAGGGCGCTGCAGCGCCTGGAGCTCCGCTACCCCCCCACCCTGGCAGCCCTGGCCCAGGAGTTGGGGGCAATGGCGGCCCGAGCCCGGCCCCCCGGCTTGCTGCTGCTGGACGGGCTGGAGCACTACATCCAGGGGGGGCCCAGCGCCCCCGCCCGCCTGGCCGCCCTGCTGCTGGAGGCCTCCCGCGCCCCCCGACCCCCGGCCCGGCTCCTGGCTGCCCTCCGTGTGCCCCCCCCCGGGCCCCGTGTGCTGCCCGTCCTGCGCCGTTACTTCCCGGCTGAGTGCCGTCTGAGACCCCTCCCCGGGGTCCCACTGCAGGTGAACGTCCGCCTCGTCCTGCCCGGATCGGTGCCGCGGGGATGGAGGCTGCGCTTCGAGCCCCACGGGGGGCTGAACGTCACCCCCGGGCCTGGGGACGGCGATGGGGACGAGGATGAGGACGGAGATGGCAGTGGGGATGAAGGCTGA

>Gallus gallus - processing of precursor 7, ribonuclease P/MRP subunit (S. cerevisiae) (POP7), CDS - 5' truncated

GGGTCCCGAAATCCGCCTCCACGGGTTGGGTTTGGCCGTCACCCGCGCCATCAACCTGGCCCTACAACTGCAGGCGGCCGCTCCCGGCGCCCTACAACTCCATGCCAGCACCTCCTCCGTCACCCTGCCCGGACGGGGGGGCAACGGGGAACCCCCCCCCCCCCACCCCCACCACCCCCACGACGATGAGGACCCCGATGCCCCCCCCCGGGACGACTCCCCCGACGCCGCTGCCCTTCGCCCCCGGCACAACTCGGCCATCCACATCCGTGTGTGCCGCGAGGCGCCGTGCGTCTGA

>Haliaeetus leucocephalus - processing of precursor 7, ribonuclease P/MRP subunit (S. cerevisiae) (POP7), CDS - internal fragment

TACGGAACCGGAGCCGACGTTTTCGTGACAGCGCGGACAGATTTCCGAGCTCAACTCCGACGTTGCCAACGTCTCCTGGCTCCAGGGGCGCCCGGGGGAGTTGGGGGATCTCGGGGGGGTCCCCCCGTCGTCCCCCCCGGGGAATTACGCCTCCACGGGCTGGGCCTGGCCGTCCCCCGCACCATCAACCTGGCCCTGCAGCTGCAGGCTGGGGCTCCCGGCGCCCTTCGGCTTCACGCCAGCACCTCCTCCGTCACCCTGCCCCCCCTCGCCACCCCCCGCTGCCGCCGGGGACCCCCGCTGGGCCGGGGTGTGGGTGAGGAGGAAGAGGTAGAAGGGTTGGAGGAGGAGGAGGAGGAAGACGGGGACGGAGCATCCCCCCGCCTCCGTCATAACTCGGCCATCCATATCAGGGTGTGCCGAGAAGCCCCCTGCGCTTGAGGTGAGGGCGCCCCGGGGT

>Gallus gallus - alkB, alkylation repair homolog 7 (ALKBH7), CDS - internal fragment

TGGGGCCGGGGGGGCGCTCTGGGGTCAGAGGTCATCGAGTCAGAGGTCACCCCCGGGATGGGGCCATTTCCGGGTTCCGGGAGGCGGAGCGCAGCCGTTGGGGGGCGCTGTCGGGGGCGGTGCTGCAGCGGATCTCCTCCGCGTTTCCCCCCGCGCGGCCCCCACTGCCCCACAGCCACATCCTGGACCTCGCGCCCCACGGCTGCGTGCGGCCCCACATCGACAGCACCAAGTTCTGTGGCTGCACCATCGCGGGGCTCTCTCTGCTGTCGGCGGCGGTGATGCGGCTGCGCAGCGTTGGGGACCCCCAGGAGTGGGCAGAGCTGCTGCTGGAGCCGCTCTCCCTTTACGTCCTGCGGGGTGAGGCGCGCTATGGGTTCACCCACGAGGTTTTGGGGGGGGAGGAGTCCTTTTTCGGGGCCCTCCGCGTCCCCCGGCAGCGCCGATTGGCCGTCATCCGCCG

>Gallus gallus - biliverdin reductase B (BLVRB), CDS

ATGGATCGCGATCGGATCGTGGCGCTGTTCGGGGCCACCGGGAGGAGCGGCCGGGAGGCGCTGCGGAGGGCGCTGCGGGAGGGCTACGCGGTATCGGCTCTGGTTCGGAACCCGGCGCTGCTGCCGCCCGACGCCGCGCCGTGCCGGGTGGTCCGCGGGGACGCGCTGCGCGCCGCCGACGTCAGCGCCACCGTGCGGGGGCAGCGCGCCGTCATCGTCACGTTGGGAACGCGCGGAGACATCGGTCCCACCACCGTCCTATCAGACAGCACCCGCAACATCGTGGCCGCCATGAAGGAGCACGGCGTGCGCAAAGTGGTGGCGTGTCTGTCCGCCTTCCTCTTATGGGATCCTGAGAAGGTCCCCACGCGGCTGCGGGCGCTGACGGAGGACCACGCGCGGATGCACGCCGTGCTGAGCGGGGCCGGGCTGGATTACGTGGCCGTCATGCCGCCCCACATCGCCGACGACAAGCCGCTGACGGAGGCATACGAGGTCACGGTCGGTGGCACCGGCGGTGGCTCGCGGGTCATCTCCACGCCGGACCTGGCCCATTTCCTCGTGCGCTGCCTCAGCACCACCGCGTTCGACGGGAAGAGCGTCTACGTCTGCGGGCACTACGGCTGA

>Gallus gallus - INO80 complex subunit E (INO80E), CDS

ATGAACGGAGCGGCGGATCCCGATGGCGGCGGAGGGAGCGGTTGCCGGCGGCGATACCGCGCCTTGAAGCGGCGGCTGCGGCTGCTGCTCTACGAGCAGGAGTGCTTCCAGGAGGAGCTGCGCCGCGCTCAGCGCCGATTGCTGCGCGTCTCCCGGGATAAGAGCTTCCTGCTGGACCGCCTGCTGCAGTACGAACACGTGGACGACGACTCCTCAGATTCCGACGCCACCGCCTCCTCCGACAGCGATGGGGAAACGCCCAAAGGGGCGGAGCCGCCTCCCCTTAAAAGGAAGCGCAGCCCCACGGGGGGCGGAGCCTCCCCACCGCCCGCCCCCGGCCTGGCCCCGCCCACTTCCTACTTGAGCACGCTGGCCTCCCCCCCATACaGCCCcTTCCCGGCTGATTACCTGGCCCCCCCCGAGCGGCCCCGCGGCCCCACACGGCGCAATAAGGGATCCCGGCGCCTGCAGCTCCCCGCCGCCCCCCCCCCGGCGCTGCCTTTCCCCCCTCCCCGCGTTTTGGGGGGGGGTTCCGTGGCCGCCCCCCCGCCCCCCCCCAAAGCCCCCGGCCCGGTGCCCCACACCGTCCCACGGCGTCTGCTCAGCGATGGGGGGGACGGCAGCGGAGACGATGGCATGGATGGGGACGACGAGCTCGTCATCGACATCCCCGAGTGA

>Gallus gallus - NADH dehydrogenase (ubiquinone) 1 beta subcomplex, 7 (NDUFB7), CDS

ATGGGAGCTCACCTGGCGCGGCGCTATGCCGGGGGGGCGGACACGGAGCCGGACCCGTTGGCGATGCCCACATTCCCCGCCGATCTGGGGCTGCCCGGCAGGGAGCCGCGCACCATGGTGGCGACGGCGCAGCAGCTGTCGGAGGGCCGCGTCCCGTTGGCTCAGCGCGACTTCTGCGCCCACCACCTCCTGCGCCTCATGCGCTGCCGCCGCGACGCCTTCCCCAGCCTATGGCACTGCCACCACCTGCGGCACCGCTGGGACCGCTGCCAGCACGACGATTATGTGATGCGCATGAAGGAGTTTGAGCGCGAGCGGCGGCTGCTGCAGCGTCAGAGGCGATCCGGGAGCGGGAGGCGGCCGTGGCTGCAGAGTGA

>Gallus gallus - 5-oxoprolinase (ATP-hydrolysing) (OPLAH), CDS

ATGGGTCCCAGCAGTCCCTATGGGGCCaATGCGTCCCCTATGGGGCTGAGGTCGGTGTGTGCCCCACAGAGCGGGGTGGCGGTTCCGGAGCGGGGCCCGCTGGACTCCCGACACGTGCAGTGGAtTCCGGATGGGGCCGAGTGcTCCtCtAtggGgcCCaGCAGtcCCtatGGGgcCGagtGctcCTcTATGGGTCcCAGCASCGRCTTCCGGGACCTTCTGCACATCGGCACCCAGGCCAGGCCCGACATCTTTGACCTGACGGTGTCGGTGCCCCCCCCGCTGTACGAGGCGGTCATAGAGGTGGACGAGCGGCTCATCCCGGCGCAGCCGCACTGCCGTCTGCCGGGCGCTCAGCGCGGGGAGCGCCGCACCGGTCGCAGCGGGGACGAAGTGTTGGTGCTGCGGCGCCCGGACGTGGAGGCGCTGCGGGCGGAGCTGCAGAAGGTGTGGGAGCGCGGAGTGCGCAGCGCCGCCGTCCTTCTGCTGCACTCCTACACCTGCCCGGATCACGAGGCTGAGGTGGGCTCCCTGCTGAGCTCCATGGGTTTCCGCCACGTGTCGCTGTCGTCGGCGCTGTCGGCCATGGCGCGCGCGGTGCCGCGGGGGATGACGGCATGCGCCGATGCGTACCTCACCCCCTGCCTGCAGCGCTACCTGCGCGGCTTCTGCCACGGCTTCAGCGACGGCCTCCAGGGGGTCCCGGTGCTGTTCATGCGCTCGGATGGGGGGCTGACCCCCATGGCGCAGTTCAGCGGTGCCCGCGCGGTGCTTTCCGGGCCGGCGGGGGGCGTTGTGGGGTACAGCCGTACCGCGGGGGGGCTCCGGGAACAGCGGCCCGTCATTGGCTTCGATATGGGAGGGACGTCGACGGACGTGAGCCGCTTTGCGGGGCGCTTGGAGCACATCTACGACGGCGTCACGGCGGGGGTCTGCATCCAGAGCCCACAGCTCGACCTGCACACCGTGGCGGCCGGGGGGGGGTCCCGTCTCTTCTACCGTTCCGGTCTCTTTGTGGTCGGCCCCGAATCCGCGGGCGCAAATCCCGGCCCCGCGTGTTACCGAAAAGGCGGCCCGGCCACGGTGACGGACGCCAACCTGGTGCTGGGCCGCCTCCTGCCCGCCTTCTTCCCGCACATCTTCGGGCCGTCGGAGGACCAACCGCTGAGCCTGGAGGCCGCCCGCAGCGCCCTGCGGGACCTGGCGGACGCCGTGGCAGCCGACGGCCACGAGGGGGCGCCGCTGAGCCTGGAGGAGGTCGCCATGGGCTTCGTCCGCGTGGCCAACGAGGCCATGAGCCGCCCCATAAGGGCGCTCACCGAGGCTCGGGGTCACCCCGTGGGGCAGCACATCCTGGCGTGCTTTGGGGGCGCGGGAGGGCAGCACGCGTGTGCCATCGCACGGGCCCTGGGCATGGACAGCGTCTTCATTCATAAACACAGCGGGGTGCTGTCGGCCTTCGGGCTGGCGCTGGCCGATGTGGTGCACGAGGCTCAGGAGCCGTCGGCGCTGCGCTACGAGGCGGCCGCGTTCGCCGCGTTGGATGAGCGCGTGGAGGCGCTGCGGGAGCGCTGCTGCGCCGCGCTGCGGGAGCAGGGATTCAACAGCTCTCAGATCCAGACGGAGCCGTTCCTCCACCTGCGCTACGCGGGGACGGACTGCGCCCTGATGTGCTCCGCTGTGGGGTACCCCCCGACCCCAAATTCCTGCCGCGCCGGGGACTTCGGGGCCGCCTTCGCTGAGCAATACCGCACGGAGTTTGGGTTCACCATCCCGGACCGGGCGGTTCTGGTGGACGACATTCGGGTTCGGGGGGTCGGCAGCAGTGGGGTCACCGAGGAGACCCCAAACCCCAGAAGGGGGGAACCCTCCGGGCCGGAGACGGTGACGCGGTGCTACTTTGAGGGGGGCTTTCTGGACACCCCGGTATTTCTGATGGAGGGGCTGAGCTGTGATCACCCCCTTCCCGGCCCCGCCATCATCATCGACCGCCACAGCACCATCGTGGTGGAGCCGGGCTGCGTGGCGGAGCTGACGCCTATGGGGGACATCCGCATCGCCGTGGGGCGCCCGACCCCACTGGTTGTGGGGCCGCAGCTCGACCCCGTGCTGCTCTCCCTCTTCTCACACCGCTTCATGAGCATCGCAGAGCAGATGGGGCGCGTCCTGCAGCGCAGCGCCATCTCCACCAACGTGAAGGAGCGCCTCGACTTCTCCTGCGCCGTCTTCGGGGCCGGCGGCGAATTGGTGTCCAACGCGCCCCACATCCCCGTGCACCTGGGGGCCATGCAGGACGCCGTGCAGTTCCAGATCCGCAGTGTTGGTGCTGATCTGCAGCCTGGGGACGTCCTGCTGAGCAACCACCCCATAGCAGGGGGCAGCCACCTCCCCGACCTCACCGTCATCACCCCCGTGTTCTGGCCGGAGCTGTCGGCGCCGGTGTTCTGGGTTGCCAGCCGGGGGCACCATGCGGATATTGGGGGGCTGACGCCGGGTTCGATGCCCCCCCATTCGAAGACCCTGAGTGAGGAGGGGGCCGTCTTCATCTCCTTCCATCTCGTGAGGGCCGGAGTGTTCCAGGAGGAGGCGGTGTCGGCGGTGCTGCAGGAGAGCGGTACGCGGGCGCTGCGGGACAACGTGGCGGACCTCCGAGCTCAGGTGGCCGCCAACCACAAGGGGGCGACACTGCTGCGGGAGTTGGTGGCCGCCTATGGGCTGAGCGGCGTCACCGCCTACATGGAGCACATCCGGGCGAACGCGGAGCGGTCGGTGCGGGAGATGCTGCGGGGGGCGGCGCGGCGCTGGGGGGCGGTGATGGAGGCGGAGGATCGAATGGACGACGGGACCCCCATCTGCCTGCGGGTCACTGTGGACCCCACTGAGGGCAGCGCAGTGTTTGATTTCTCGGGTTCGGGCCCGGAGGTTTATGGGAACTGCAACGCCCCGCGGGCCATCACACTGTCAGCCCTCATCTACTGCCTGCGCTGCATGGTGGGCCACGACATCCCCCTCAACCAGGGCTGCCTGGCCCCGGTGCGGGTGCTCCTTCCGGAAGGTTCCATCCTCAGCCCGTCCCCGCAGGCGGCGGTGGTTGGGGGCAACGTGCTGACATCGCAGCGCATCGTCGATGTGGTGCTCAGAGCCTTCGGGGCCTGCGCGGCGTCGCAGGGCTGTATGAACAACGTGACGTTCGGCGACGCGTCCATTGGTTACTACGAGACGGTGGCGGGCGGGGCCGGCGCGGGGCCGCATTGGGCCGGGCGCAGCGGCGTGCACAGTCACATGACCAACACGCGCATCACCGACCCCGAGATCCTCGAGCTGCGTTACCCGGTGGTGGTGCGGCGCTTTGAGCTGCGCCGCGGTTCGGGCGGTTCGGGGCGATTCCGGGGCGGTGACGGAGTGAGGAGAGAGCTGCAGTTCAGAGCCCCCCTCGTGCTGTCCGTGCTCAGCGAGCGCCGCGTCACGCAGCCCTACGGCATGCAGGGCGGAGCTGCGGGCGCCCGCGGGGTGAATCTGCTGCAGCGCTGCGACGGCCGCGTGCTCAGCCTGGGGCCCAAAGCCTGCGTCAGCGTGGGGCCGGGGGACGTATTCATCCTTCTGACCCCCGGCGGTGGCGGCTTTGGGACCCCCGAGGAGGATGGGGGTGAAGGGGGGGCGCACAGCCCCAAACCCACCGGGGCCCGCGAGTACTGGGAGGGCACTGAGGCACACTGA

>Gallus gallus - Purkinje cell protein 2 (PCP2), CDS

ATGGGTGGGGGTCTCCCTGAGCGCCGTGTTTGCCGCGGGGCGGAGCCCCGGAGCGGGGGGGGTCTCCGGACGGGGGGTGGGTCCCCGGAGGGCCAGGAGGGCTTCTTTACCCTCCTGAGCTCCGTGCAGGGGGCCCGCATGGATGAGCAGCGCTGCAGCCTGGGGGGGGGCGGGCCCCCCCCCGAGCTGGCCACCCTGCTGGATTTGGTCGCCCACTCTCAGGGCCGCCGATTGGACGAGCAGCGCCTGGGGGTGCAGCGGCTGCCGGGTTTTGGGGGGCCCCCCCCGGATGGAAGCACTGCGAGCGGGGATGGGAA

>Gallus gallus - PET100 homolog (PET100), CDS

ATGGGGGTGAAGTTGGAGGTGTTCCGGATGCTGCTGTACCTCTCGTTCCCCGTCGGTGTCTTCTGGGTGTCCAATCAGGCTCAGTACTTCCAGCAGTTCGTCGTGCAGCGCAGGAGAGAGATCTTCCCTCCGGACAACCCCGAGCGGCGCCGTGAGGTGGCGGCGCTGAAGCAGCGGGTGCTGCGGATCCAGGAGGAGCGCGCGCTGCGGGACACGCGTGGGTAG
