# Supplementary material for: Hidden genes in birds
Source: Genome Biol. 2015 Aug 18;16(1):164. doi: 10.1186/s13059-015-0724-z (PMC4539667; doi:10.1186/s13059-015-0724-z)
Supplement: Additional file 2: — Phylogenetic analysis of LPPR2, MMP14, and MRPL52. The three newly assembled genes were analyzed together with orthologous sequences from other vertebrates, and with their closest paralogs, if those were available. The list of GenBank sequences used in the alignment is given bellow. Amino acid sequences were aligned with the MUSCLE algorithm and uninformative regions were removed using trimAl v1.4 software. The maximum likelihood (ML) phylogeny was constructed in MEGA 6.06 software, using the JTT substitution model, Nearest-Neighbor-Interchange ML heuristic method and otherwise default parameters. Support for the ML tree was assessed by 200 nonparametric bootstrap replicates. Bootstrap values higher than 0.95 are shown. Red asterisks mark the newly assembled avian genes. Scale bars show the number of amino acid substitutions per site. [file 13059_2015_724_MOESM2_ESM.pdf]

Additional file 2

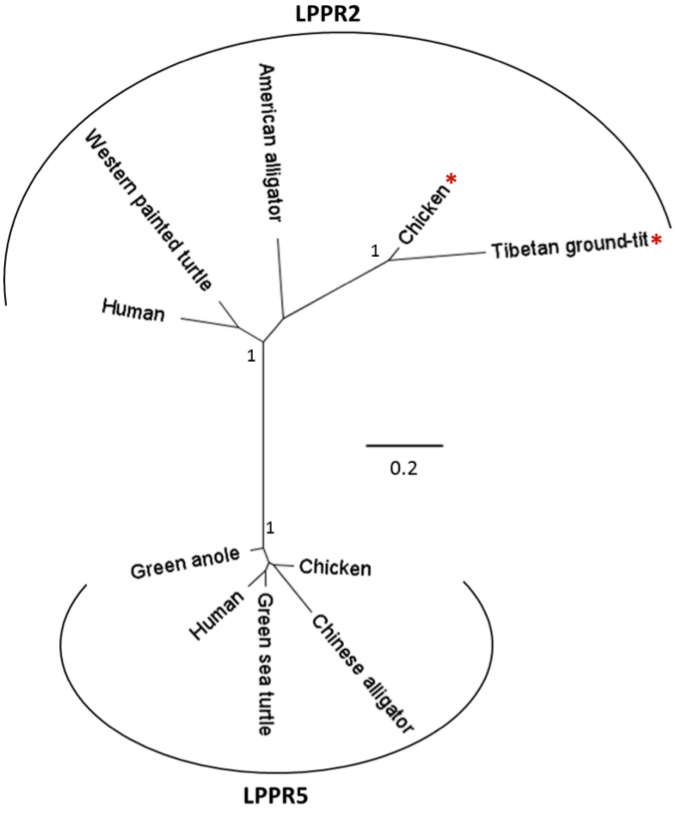

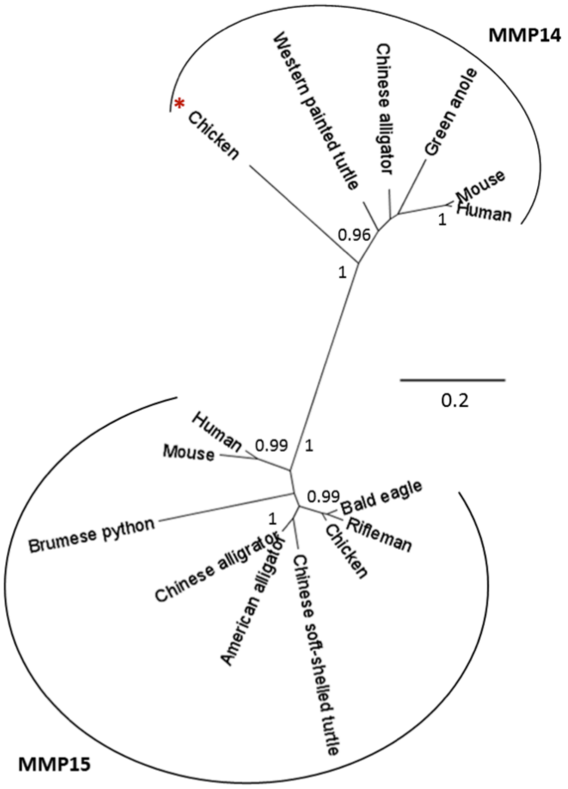

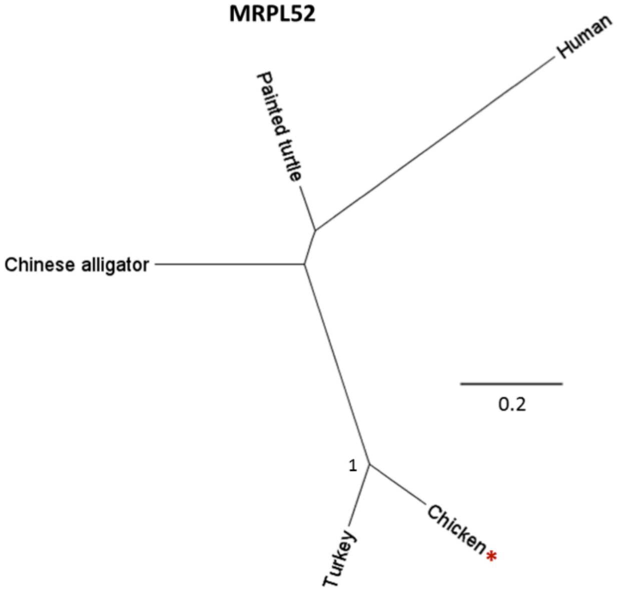

Gene	Organism	Gene ID
LPPR2	Homo sapiens	282400943
LPPR2like	Alligator mississippiensis	564246552
LPPR2like	Chrysemys picta bellii	530660999
LPPR5	Gallus gallus	513196992
LPPR5	Chelonia mydas	591375973
LPPR5	Anolis carolinensis	637292948
LPPR5	Alligator sinensis	557258930
LPPR5	Homo sapiens	82659092
MMP14	Mus musculus	188528636
MMP14	Homo sapiens	526479827
MMP14	Chrysemys picta bellii	530593169
MMP14	Anolis carolinensis	637354335
MMP14	Alligator sinensis	557328456
MMP15	Mus musculus	55926148
MMP15	Gallus gallus	513202660
MMP15	Haliaeetus leucocephalus	729724462
MMP15	Acanthisitta chloris	677986855
MMP15	Python bivittatus	602669425
MMP15	Alligator mississippiensis	564265923
MMP15	Pelodiscus sinensis	558185053
MMP15	Alligator sinensis	557270018
MMP15	Homo sapiens	45594662
MRPL52	Meleagris gallopavo	733928499
MRPL52	Alligator sinensis	557328460
MRPL52	Chrysemys picta bellii	530593173
MRPL52	Homo sapiens	169646288
