# Supplementary figures and images for: Hidden genes in birds
Source: Genome Biol. 2015 Aug 18;16(1):164. doi: 10.1186/s13059-015-0724-z (PMC4539667; doi:10.1186/s13059-015-0724-z)

## Additional file 3

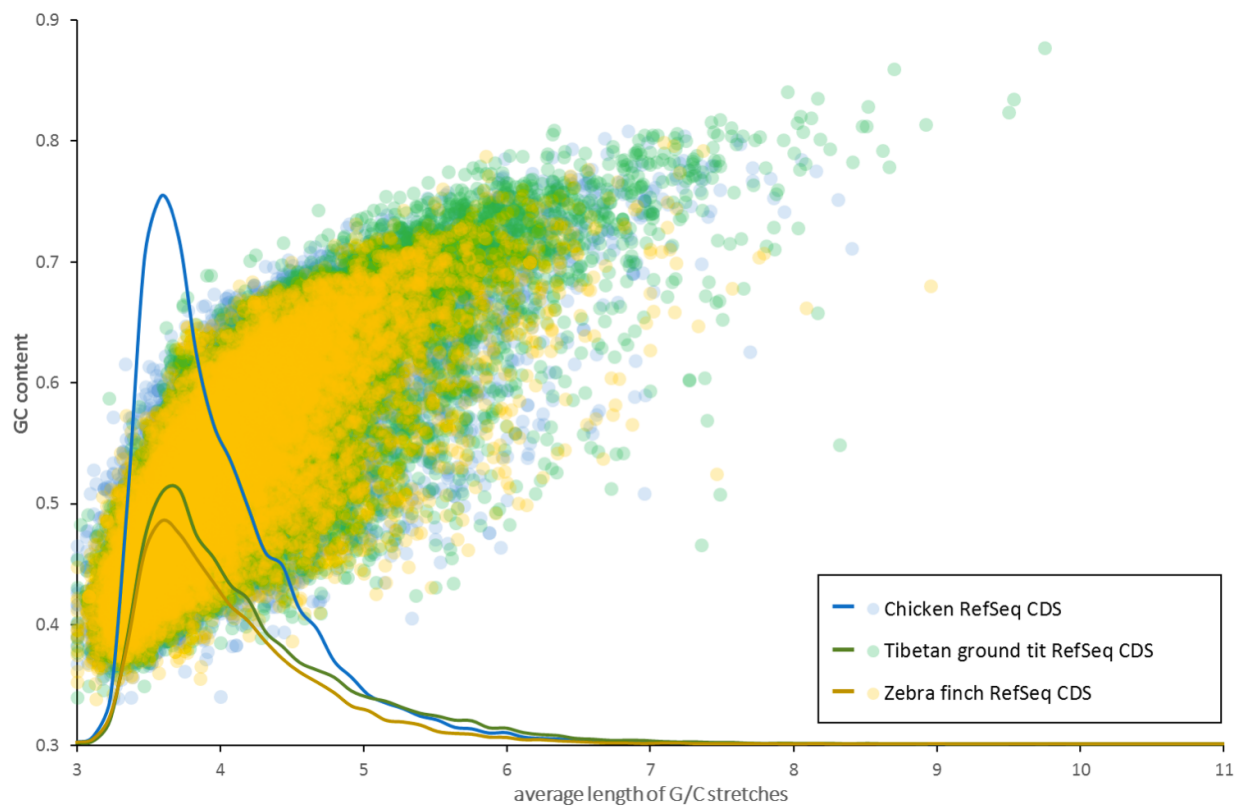

Supplement: Additional file 3: — Comparison of GC-content and the presence of G/C stretches in genes of three avian species. The GenBank RefSeq datasets for chicken (Gallus gallus), Tibetan ground tit (P. humilis), and zebra finch (Taeniopygia guttata) were analyzed. Only coding sequences with length greater than 299 nucleotides were included. GC-stretch was defined as in legend to Fig. 1. Both dot plots and histograms of G/C stretch average lengths are shown. The histograms have similar shapes but not heights, because different number of genes is annotated in the three avian species. [file 13059_2015_724_MOESM3_ESM.pdf]
