# Supplementary material for: Hidden genes in birds
Source: Genome Biol. 2015 Aug 18;16(1):164. doi: 10.1186/s13059-015-0724-z (PMC4539667; doi:10.1186/s13059-015-0724-z)
Supplement: Additional file 4: — List of avian genes and their vertebrate orthologs used in Fig. 1b . [file 13059_2015_724_MOESM4_ESM.pdf]

Additional file 4

Gene	Organism	Gene ID
EPO	Anolis carolinensis	637379669
EPO	Homo sapiens	62240996
EPO	Pelodiscus sinensis	558123633
EPO	Mus musculus	113931667
EPOR	Pelodiscus sinensis	558169966
EPOR	Chrysemys picta bellii	641780587
EPOR	Chelonia mydas	591366899
EPOR	Alligator mississippiensis	564246600
EPOR	Homo sapiens	296040523
EPOR	Mus musculus	568958822
EPOR	Python bivittatus	602632393
EPOR	Anolis carolinensis	637264122
LPPR2	Chrysemys picta bellii	530660999
LPPR2	Homo sapiens	282400943
LPPR2	Alligator mississippiensis	564246552
LPPR2	Anolis carolinensis	637264541
LPPR2	Python bivittatus	602632389
LPPR2	Mus musculus	592743502
MMP14	Chrysemys picta bellii	530593169
MMP14	Alligator sinensis	557328456
MMP14	Homo sapiens	526479827
MMP14	Anolis carolinensis	637354335
MMP14	Mus musculus	188528636
POP7	Pseudopodoces humilis	543381286
POP7	Chrysemys picta bellii	530660816
POP7	Alligator mississippiensis	564247760
POP7	Pelodiscus sinensis	558123640
POP7	Homo sapiens	153791430
POP7	Mus musculus	209954833
SWSAP1	Mus musculus	244791056
SWSAP1	Pseudopodoces humilis	543382615
SWSAP1	Chrysemys picta bellii	530647204
SWSAP1	Chelonia mydas	591366901
SWSAP1	Anolis carolinensis	637264118
SWSAP1	Pelodiscus sinensis	558169970
SWSAP1	Homo sapiens	190684678
SWSAP1	Python bivittatus	602632391
